# Ionic Liquids as Starch Plasticizers: The State of the Art

**DOI:** 10.3390/molecules30051035

**Published:** 2025-02-24

**Authors:** Susanna Romano, Serena De Santis, Chiara Frezza, Monica Orsini, Giovanni Sotgiu, Marta Feroci, Daniele Rocco

**Affiliations:** 1 Department of Industrial, Electronic and Mechanical Engineering, Roma Tre University, via Vito Volterra, 62, 00146 Rome, Italy; susanna.romano@uniroma3.it (S.R.); serena.desantis@uniroma3.it (S.D.S.); chiara.frezza@uniroma3.it (C.F.); monica.orsini@uniroma3.it (M.O.); giovanni.sotgiu@uniroma3.it (G.S.); 2 Department of Fundamental and Applied Sciences for Engineering, Sapienza University of Rome, via Castro Laurenziano, 7, 00161 Rome, Italy

**Keywords:** ionic liquids, plasticizers, starch, imidazolium, choline-derived bioIL

## Abstract

Over the past 15 years, ionic liquids (ILs) have gained increasing attention as potential replacements for traditional organic compounds. Thanks to their remarkable properties, such as non-volatility, chemical stability, low toxicity, solvation power, and the tunability of properties—due to different combinations of cations and anions—ILs are considered ideal in the processing of polymers. Indeed, they have been extensively studied for the dissolution, derivatization, and plasticization of biopolymers to address the growing issue of plastic pollution. The aim of this review is to investigate the recent years’ literature using ILs in starch plasticization. In particular, two major classes of ionic liquids were addressed, the imidazole-based ionic liquids and the choline-derived bioILs. Furthermore, this review aims to provide a comprehensive understanding of the mechanisms behind the interactions between ILs and starch and to study their effect on biopolymer properties.

## 1. Introduction

Non-volatility, non-flammability, low viscosity, chemical and electrochemical stability, negligible vapor pressure, conductivity, solvating power, and low toxicity are just some of the outstanding properties of ionic liquids (ILs) [1,2]. In view of the simultaneous presence of all these interesting properties, it is not surprising that these salts, consisting of a large organic cation coupled with a non-coordinating organic or inorganic anion, were involved with completely different roles in a large number of application fields. Organocatalysis [3], electrochemistry [4], separation and purification methods [5,6,7], energy applications [8], and cultural heritage protection [9] are only some of them. In addition, if their ability to form hydrogen bonds simultaneously with the capability of dissolving high molecular weight molecules, such as polysaccharides [10,11,12,13], is taken into consideration, the potential of ILs is greatly increased, as is their applications. This is because all the described physico-chemical properties are consistent with those required to be considered as plasticizers in the plasticizing processes of biopolymers [14]. The problem of pollution due to the overuse of fossil-based plastics is certainly one of the challenges of our time. Recycling plastics is surely a valid solution, but the world plan has not yet taken off fully, especially in some parts of the planet, with the unsatisfactory result that only 10% of waste plastics are recycled and more than 60% of global plastics are disposed of directly into landfills [15].

Therefore, the obligation is to find safer alternatives to synthetic plastics, and from this point of view, scientific research is not going to back out. In this respect, the development of bioplastics is a central topic and is currently considered the main way forward. Among them, starch results as one of the most promising candidates, obtainable from plant sources like wheat, tapioca, potatoes, and maize and possessing excellent biodegradability and biocompatibility properties [16]. However, the intermolecular forces and hydrogen bonds existing between polymeric chains determine the semicrystalline granular structure responsible for its poor mechanical properties and low processability with the common production techniques of plastic materials, like extrusion and injection molding [17,18]. As a result, one of the most valuable methods for rendering starch suitable consists of using plasticizers that are able to weaken starch hydrogen bonds and disrupt its 3D structure to obtain a homogeneous amorphous material named thermoplastic starch (TPS) [18]. Therefore, an ideal plasticizer should be able to interact with starch, be safe for humans and the environment, have good chemical and thermal stability in addition to low volatility [19]. From a strictly chemical point of view, the choice falls on species capable of forming hydrogen bonds, replacing those already present between starch chains, with the ultimate goal to decrease the glass transition temperature *Tg* and improve the starch chain mobility [20,21]. Water, polyols (glycerol, glycol, sorbitol, etc.), citric acid, and urea were the first starch plasticizers but all of them possess only some of the above-mentioned features, forcing the researchers in the field to find other solutions. Some of them have decided to focus on ILs. The IL properties described above are fully in line with plasticizer ones, with the peculiarity that in the case of ILs, the latter can be tuned as necessary thanks to the infinite choice of possible ion pairs. It should be highlighted that despite the wide variety of plasticizers employed, much of the current research makes use of the solution casting method, although the process is not scalable. To date, there is limited research on the applicability of ionic liquids in industrial processes. However, the most recent studies are already examining the effects of ionic liquids on starch extrusion, albeit still on a laboratory scale [22].

However, it is worth taking into account that some of their characteristics, such as their high solubility in water, may lead ILs to have a significant impact on living organisms. Indeed, to date, there is a limited number of studies on their potential toxicity. Nevertheless, it is highlighted in the literature that the main structural factor determining cytotoxicity is the alkyl side chains of the cation, while the impact of the anion is lower, especially the chloride [23].

The purpose of this review is therefore to present the research works concerning the use of ILs as starch plasticizers. The reviewed scientific articles have been reported on to shed light on how the authors demonstrated the interaction between starch and plasticizers according to the considered characterization technique. Consequently, the effects of the interaction with ILs on the properties of the crystallinity, thermal stability, electrical conductivity, and Fourier transform infrared spectroscopy (FTIR) response of the starch are shown.

Additionally, to the performances shown by the common imidazolium ILs, a part is devoted to the description of the efficiency as starch plasticizers of bioionic liquids (bioILs) based on a choline cation.

### 1.1. Starch Plasticized by Imidazolium Ionic Liquids

**Figure 1:**
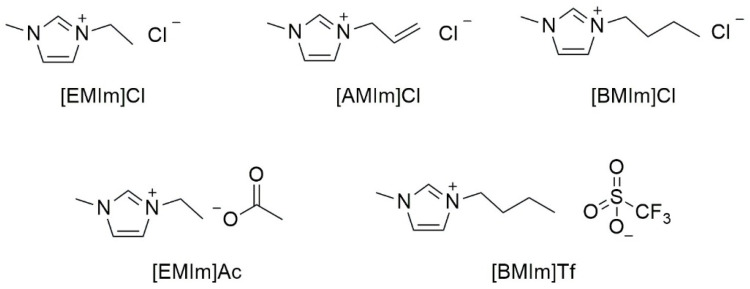
Structures of imidazolium ILs considered in this review.

Figure 1 reports the structures of imidazolium ILs, which have been used as starch plasticizers in the scientific publications considered in this review.

**Figure 2:**
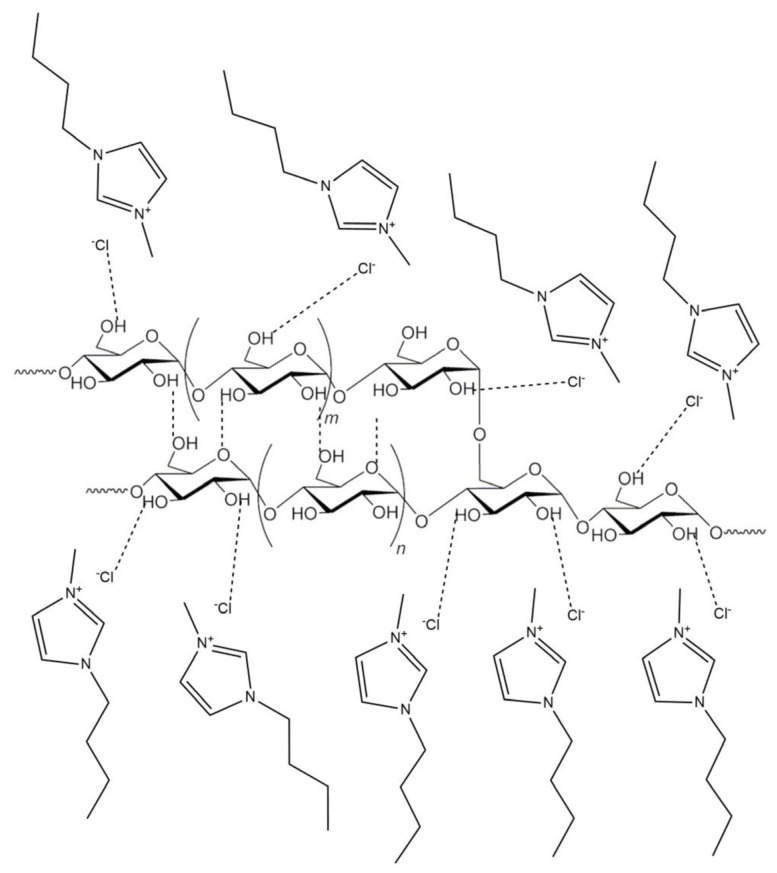
Hydrogen bonding between starch and 1-butyl-3-methylimidazolium chloride. Ref. [24] © 2020 by the authors. Licensee MDPI, Basel, Switzerland. This article is an open access article distributed under the terms and conditions of the Creative Commons Attribution (CC BY) license.

When imidazolium ILs are considered, the possibility of hydrogen bonding with starch seems to be mainly due to the anionic part of the IL and to a lesser extent to the imidazolium cation [24], as reported in Figure 2.

**Figure 3:**
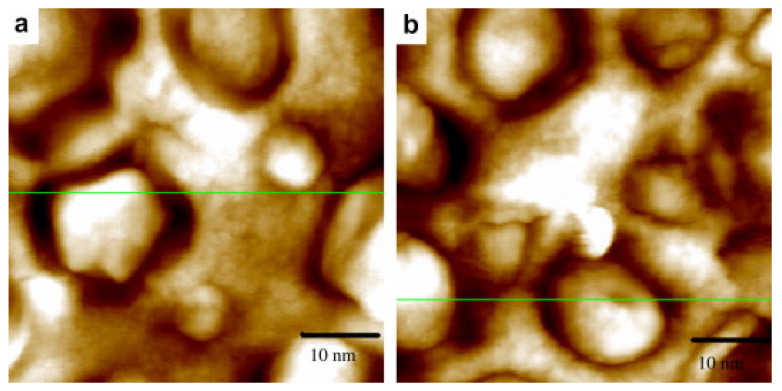
AFM images of (**a**) glycerol/TPS and (**b**) [AMIm]Cl. Reproduced under terms of the CC-BY license [25]. Copyright © 2008 Elsevier Ltd. All rights reserved.

Using the solvent casting method, Ning et al. prepared several conductive corn starch-based films with glycerol and 1-allyl-3-methylimidazolium chloride [AMIm]Cl as plasticizers [25]. For the purpose of conducting a comparative study, the authors formulated some proportional glycerol/[AMIm]Cl mixtures, starting from glycerol and gradually increasing the IL content. In the first instance, atomic force microscopy (AFM) was chosen to evaluate the plasticization of native starch by defining the average diameter of residual starch granules. The AFM images (Figure 3) showed a similar starch particle size (10 nm) incorporated in the thermoplastic starch matrix (TPS) in the cases where glycerol and [AMIm]Cl were alone and therefore more comparable.

**Figure 4:**
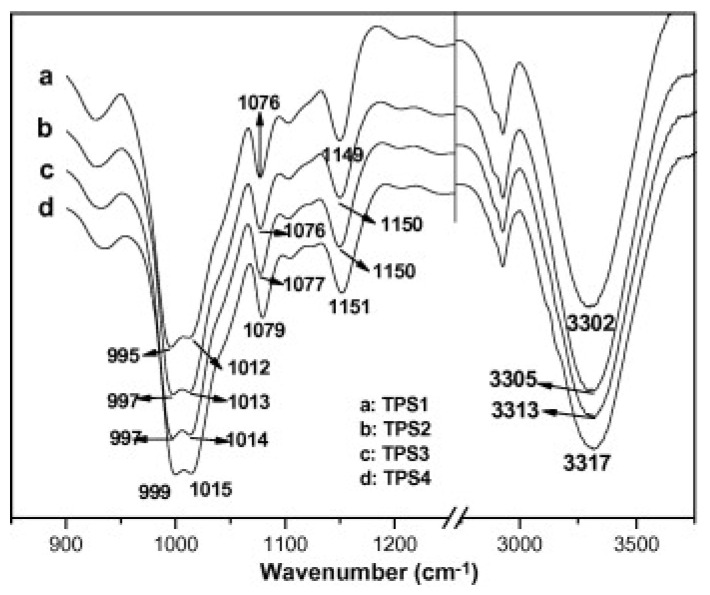
FT-IR spectra of TPS films. TPS1 contains only glycerol. TPS4 contains only [AMIm]Cl Reproduced under terms of the CC-BY license [25]. Copyright © 2008 Elsevier Ltd. All rights reserved.

Certainly, the AFM microscopy revealed an interaction between the IL and the starch, showing the presence of homogeneous TPS. Consequently, availing of FT-IR spectroscopy (Figure 4), the type of interaction survey was performed, focusing the attention on the peak frequency of starch C-O group and following the rule for which the frequency decrease coincides with greater interaction between starch and the plasticizer [26,27]. As a result, the shift in the peak positions to higher frequencies, increasing the [AMIm]Cl content, suggested less of a hydrogen bond-forming ability of the IL than glycerol.

Nevertheless, [AMIm]Cl-plasticized-TPS films showed interesting conductance values compared to glycerol-plasticized-one, proving to be an excellent compromise between the interaction ability with the starch and the possibility to obtain electroactive polymers. Therefore, for the first time, an IL shall be considered as a plasticizer to produce TPS.

Subsequently, Sankri and co-workers investigated the plasticization of the native starch using 1-butyl-3-methylimidazolium chloride ([BMIm]Cl) as the IL [28]. In this case, the authors compared not only two different plasticizers, an IL and glycerol, but also two different processing scales: a twin-screw extruder to evaluate the practicability of the [BMIm]Cl on the industrial scale and a micro compounder for laboratory scales. An initial macromolecular structure characterization revealed similar molecular weights for both cases of starch/[BMIm]Cl and starch/glycerol extruded samples, with slightly smaller values in the IL plasticizer case. The small difference was attributed to the small derivatizing effect of [BMIm]Cl. Although the hydrophilic behavior of the IL [29] surprisingly TPS/[BMIm]Cl showed lower hydrophilic properties than the TPS/glycerol sample. The authors explain this incongruity as the presence of a particular interaction between [BMIm]Cl and starch, which would limit the interaction of the latter with water. If confirmed, this hypothesis could be a potential additional tool to study the interaction between ILs and biopolymers in general. Again, FT-IR spectroscopy proved to be a valuable tool to study the possible interaction between starch and plasticizer. Regarding the hydrogen atoms of the O-H groups, Sankri and co-workers decided to follow a different path, not being able to trust the peak positions due to the presence of the high noise. They concluded that the interactions of [BMIm]Cl with O-H groups were demonstrated observing the peaks above 3000 cm^−1^, which have an amplitude band from 391 cm^−1^ in the presence of glycerol to 447 cm^−1^ in the presence of the IL. As regards the other reference starch functional groups, such as C–O bonds in C–O–C of anhydroglucose rings and C-O-H groups, the authors have always seen an increase in the wavenumber values of the maximum absorption peaks. By invoking past research work [30], they claim that the interaction between the IL and starch takes place through hydrogen bonds involving the ion pair BMIm^+^ and Cl^−^ with the oxygen and hydrogen of the same C-O-H group of starch, respectively.

**Figure 5:**
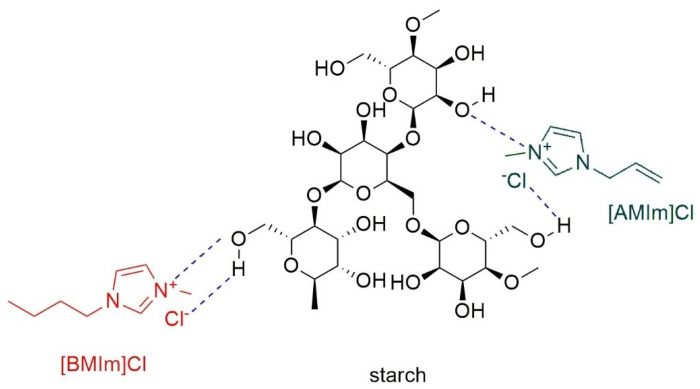
The hypothesis of the interaction mechanism between starch with [BMIm]Cl and [AMIm]Cl [28].

All this leads to an overall loss of the hydrogen bonding intensity to which TPS is submitted with an increase in the vibrational mode wavenumber values of the functional groups involved. Moreover, they suggested a different interaction mechanism in the previous case, where the other authors used [AMIm]Cl. Since Ning et al. observed a decrease in frequency related to O-H stretching at 3300 cm^−1^ increasing the IL content, [AMIm]Cl probably interacts through the Cl^−^ anion with the hydrogen of O-H groups of starch, while the [AMIm]^+^ cation weakly binds the oxygen atom of another different group (Figure 5). Consequently, starch is still able to form hydrogen bonds with water or other O-H groups of starch.

**Figure 6:**
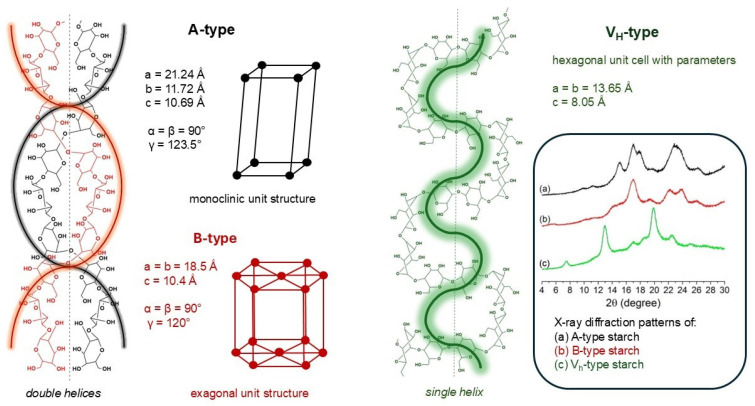
Schematic view of possible crystalline arrangements of the starch [32].

Finally, the authors showed mechanical tensile results, which confirmed a strong plasticization of starch by [BMIm]Cl. The papers described before encouraged other research groups of the field, and the use of imidazolium ILs as starch plasticizers became popular. Zdanowicz et al. investigated the [AMIm]Cl-plasticized-TPS properties performing several characterization techniques, including rheometric analysis, hot compression molding tests, and X-ray diffraction (XRD) [31]. In particular, [AMIm]Cl-plasticized-TPS XRD curves showed the reduction of the signal intensity relating to B-type crystallinity in the range of 18–20° when compared with native starch, showcasing high amorphization degree of the sample. Figure 6 shows most common crystalline arrangements of the starch. 

IL-plasticized-TPS intercepted the requirements of multiple research lines and challenges of present day, including ionic liquid-based biopolymer electrolytes [33,34]. Liew et al. decided to stake on starch and its renewable and biodegradable properties as a polymer matrix to develop a new environmentally friendly polymer electrolyte [35]. The plasticizer choice fell on the IL, 1-butyl-3-methylimidazolium trifluoromethanesulfonate [BIMIm]Tf. Differential scanning calorimetry (DSC) showed a strong plasticizing effect of [BIMIm]Tf with a glass transition temperature (*T_g_)* value of [BIMIm]Tf/TPS well below the starch native value of 56 °C (30 wt.% of [BIMIm]Tf, *T_g_* of −22 °C). Moreover, the further increase in the IL content led to a supplementary decrease in the *T_g_* values and simultaneously the improvement of the ionic conducting character of the TPS complex. Therefore, the authors observed an influence of the high plasticization ability of the IL [BIMIm]Tf, demonstrated also by the XRD analysis, on the ionic conductivity of the polymer matrix. In 2013, Bendaoud and Chalamet expanded the list of potential IL plasticizers of starch, introducing 1-ethyl-3-methylimidazolium acetate [EMIm]Ac [36]. An XRD analysis immediately showed the competitiveness of [EMIm]Ac in the thermoplasticization process of the starch not only with glycerol but even with other ILs. Despite the percentage of the residual crystallinity of the [EMIm]Ac/TPS sample being less than the [AMIm]Cl/TPS value, it was still comparable with the [BMIm]Cl/TPS one. In addition, for the formulation of [EMIm]Ac/TPS 30% (*w*/*w*), the authors defined the system as amorphous, while they were still able to calculate the crystallinity in the same formulation with glycerol. Moreover, water absorption studies have been performed in order to investigate the interaction of starch/water/starch in different experimental conditions (relative humidity percentage RH and plasticizer content). AMImCl/TPS and BMImCl/TPS showed the lowest trend in terms of water absorption among the IL/TPS samples.

**Figure 7:**
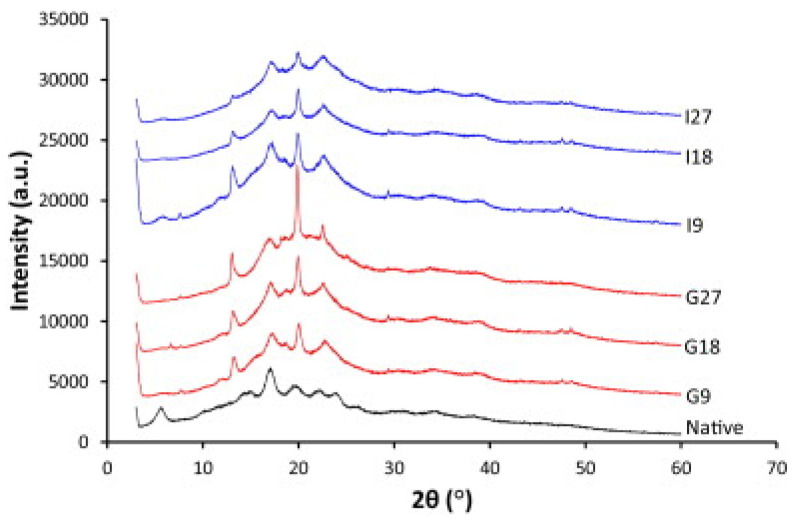
XRD patterns of native starch (Gelose 80) and the different TPS samples. The red color refers to samples with glycerol. The blue color refers to samples with [EMIm]Ac. Reproduced under terms of the CC-BY license [37]. Copyright © 2014 Elsevier Ltd. All rights reserved.

Xie et al. continued the starch plasticization studies with [EMIm]Ac, using the compression molding method to disperse the IL in the native starch and obtain different TPS samples [37]. They performed several characterization techniques, such as SEM, NMR, DMTA analysis, and so on, but the most interesting results seemed to arrive from the XRD analysis. The native starch XRD profile is characterized by the presence of diffraction peaks at *2θ* of 17° and by a series of weaker signals, which together describe the B-type crystalline structure (Figure 7) [38,39].

However, after processing, regardless of which plasticizer is used, glycerol or [EMIm]Ac, several other peaks appear related to the V_H_-type crystalline structure, a single-helical amylose structure [38,40]. Nevertheless, while the increase in glycerol content resulted in an enhancement of V_H_-type crystallinity, in the case of [EMIm]Ac, the increase in IL content led to the simultaneous loss of the V_H_-type crystallinity and B-type crystallinity. As a result, not promoting the single-helical structure formation [EMIm]Ac allowed for a major decrease in the total crystallinity if compared with glycerol, which showed an inverse trend regarding B-type crystallinity and the V_H_-type one.

The experimental results induced the same authors to investigate more specifically the starch/[EMIm]Ac interaction, preparing several samples using the same preparation technique and two different starch amylose contents [41]. Gelose 80 (G80) and regular maize starch (RMS) were used, with amylose contents of 82.9% and 24.4%, respectively. The first corresponded to the starch used in the previous work, and therefore, it showed the B-type crystallinity as described above. RMS XRD pattern was different, showing a diverse crystalline arrangement belonging to A-type crystalline structure occurring with strong peaks at 15° and 23° and an unresolved doublet at *2θ* of 17° and 18° [32,42]. However, XRD results revealed that [EMIm]Ac was able to interact with both crystalline structures because after processing of RMS samples, the two signals at *2θ* of 17° and 18° disappeared. Moreover, the final result was a similar crystalline pattern of G80 plasticized samples with newly formed V_H_-type and B-type crystalline structures.

In summary, despite the different crystal structure of the starting point, [EMIm]Ac interacted with both native starches and led to a similar final crystal structure. This would explain the similar mechanical properties, glass transition temperature, and thermal stability of G80 and RMS starch films. Consequently, later RMS starch plasticized with [EMIm]Ac was the reference polymer of the research group in the preparation of electroconductive films [43]. This choice was successful because TPS/[EMIm]Ac films displayed excellent electrical conductivity (>10^−3^ S/cm).

IL/starch interaction was now proven and the plasticizing capabilities of some ILs confirmed. Thus, first application studies in the direction of industrial demands took hold, and the possibility of using ILs as an effective solution was beginning to be tested.

For example, Liu and co-workers prepared for the first time PBS/starch blends composed of [BIMIm]Cl plasticized starch [44]. After SEM image acquisition on the studied blends, the authors claimed that the better plasticization ability of the IL than glycerol allowed for the major dispersion of the TPS in PBS. In addition, the increased polymer compatibility due to an effective plasticizing process led to an improvement in the mechanical properties as tensile strength and elongation at the break. Later, Zhao et al. used the same blends and IL to study a possible synergistic effect of [BIMIm]Cl and inorganic salts with different anions to improve these same polymer compatibilization and mechanical properties [45].

Bendaoud and Chalamet focused on other, no-less-important issues as the development of new sustainable processing techniques and green approaches [46]. They decided to combine the supercritical fluid technology (ScCO_2_) with ILs in the thermoplasticization obtained through the extrusion process of the starch using carbon dioxide as the fluid and [BIMIm]Cl as the IL [47]. The effect of the pressure, temperature, and treatment time have been investigated, comparing the experimental results of several characterization techniques with those obtained on starch plasticized with 20 wt% of glycerol. In particular, focusing on the thermal characterization, the authors observed that the effect of the different processing parameters on the *T_g_* values was more pronounced in the case of [BIMIm]Cl than the glycerol one, leading to more variety of *T_g_* values distributed over a wider range. As a result, TPS with different properties can be obtained depending on the supercritical fluid conditions adopted by exploiting scCO_2_ with the IL with respect to glycerol. Effectively, the idea of developing new processing techniques or real versatile plasticizers, allowing to tune the properties of TPS according to the demands, became widespread. Ren et al. adopted the approach of the combined plasticizers with different formulations of the glycerol/[BMIM]Cl mixture, trying to tune some essential properties of the TPS [48]. Actually, the experimental observations previously described suggested this option, especially regarding the crystallinity issue and the inverse impact that glycerol and [BMIm]Cl showed against the V_H_-type crystallinity formation growing. Moreover, the authors proved that a high content of [BMIm]Cl led to a decrease in the thermal stability of TPS. Consequently, a correct formulation of the two plasticizers allowed them to exploit the greater plasticizing ability of the IL safeguarding the thermal stability of the final starch film.

**Figure 8:**
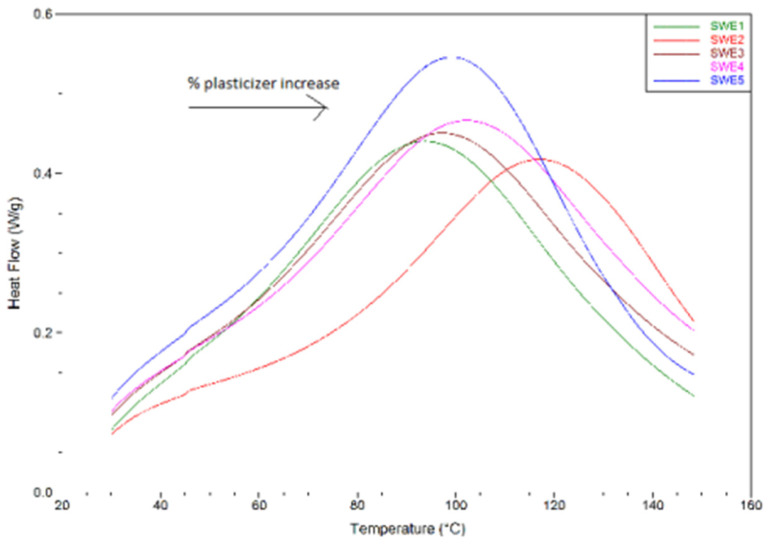
DSC curves of gelatinization for starch/water/[EMIm]Ac system. Reproduced under terms of the Creative Commons CC-BY-NC-ND license and permits [49] © 2016 The Author(s). Published by Elsevier Ltd.

Ismail et al. made use of DSC to extensively study starch/[EMIm]Ac interaction and how the latter would affect the behavior of the final TPS samples towards water [49]. A DSC analysis was carried out on several TPS samples containing different [EMIM]Ac content with the intention of extrapolating the onset temperature (*T_o_*), the peak temperature (*T_p_*), the melting temperature (*T_m_*), and the melting enthalpy (Δ*H*). All these parameters turn out to be crucial to derive information regarding the interaction with starch, all of them susceptible to the hydrogen bonding capacity of plasticizers [50]. In all examined cases, the authors observed a single endothermic transition, corresponding to the melting process and gelatinization of the starch, and extrapolated higher *T_o_* and *T_p_* values, increasing the IL content (Figure 8). Therefore, the presence of the IL would actually contribute negatively on the gelatinization process of starch, highlighting, despite the high plasticizing properties of ILs, the importance of the water contribution in the overall process. As before, a correct formulation of the two plasticizers (water and [EMIm]Ac) should be developed.

Subsequently, the same research group compared the thermal behavior of TPS plasticized by two different ILs: [EMIm]Ac and [EMIm]Cl [51]. In this paper, based on the experimental results obtained by TGA and DTG curves and comparing the peak temperature of the TPSs, a higher plasticizing ability of [EMIm]Ac was declared. At the same time, however, the authors found lower thermal stability of the TPS/[EMIm]Ac samples.

**Figure 9:**
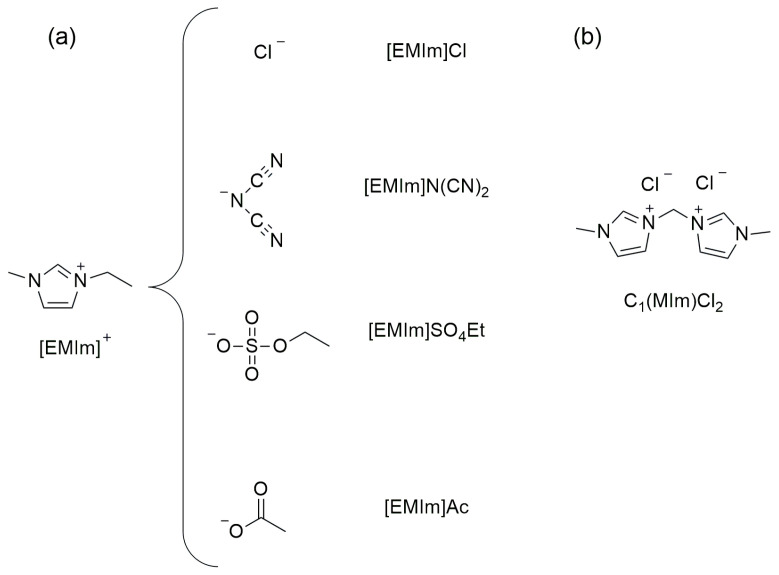
Structures of (**a**) imidazolium ILs and (**b**) dicationic imidazolium salt.

However, in general, [EMIm]Ac began to prevail over other ILs thanks to its favorite skills to interact with starch polymer chains. Following this conclusion, Domene-López and co-workers selected [EMIm]Ac as the IL plasticizer reference to prepare several TPS films starting from starch derived from different botanical origin [52]. The literature reported that generally, tuber starch contains a higher phosphate monoester concentration than cereal starch [53], and indeed, differences in the functional group content should lead to having TPS films with diverse physico-chemical and mechanical properties. In fact, the XRD analysis showed a greater degree of crystallinity of corn and wheat TPS films than those of potato TPS ones, determining higher opacity but better mechanical properties. Certainly, given the promising TPS properties and the large number of application fields that are now investing on the starch, in future perspectives, any effort to find new, different sources is essential. Abera and co-workers tested a less common typology starch obtained from anchote (*Coccinia abyssinica*), and again, [EMIm]Ac was selected as plasticizer to prepare TPS films [54]. [EMIM]Ac proved good ability to interact with Anchote starch and the resulting TPS films showed higher flexibility and better mechanical properties than those obtained from polyols (glycerol, sorbitol, and triethylene glycol). Recently, we reported the preparation of TPS films using 1-ethyl-3-methyl imidazolium-based ionic liquids as plasticizers studying the influence of different anions ([SO_4_Et]^−^, [N(CN)_2_]^−^, [OAc]^−^, and [Cl]^−^) on the morphology, crystalline structure, glass transition temperature, thermal stability, and electrical conductivity (Figure 9) [55]. All characterization techniques (DSC, XRD, FT-IR, and mechanical tests) proven the plasticizing effect of all ILs, although [EMIm]Ac turned out to be the best IL to interact with starch. Regarding the crystallinity, [EMIm]Ac and [EMIm]Cl led to the same crystal structure after recrystallization, with weak XRD signals related to V_H_-type comparing with B-type ones. Finally, a first attempt of the use of a dicationic imidazolium salt (DIL) as plasticizer of the starch was performed, obtaining promising experimental results. These salts possess a bidentate nature and more different structural combinations compared with their corresponding monocationic ones, also having the variable of the spacer chain [56]. As a result, DILs may open endless possibilities of new plasticizers, not only for starch but for biopolymers in general. DSC traces the gelatinization for the starch/water/[Emim]Ac system.

Table 1 summarizes the properties and characteristics of the different types of starch plasticized by imidazolium ionic liquids. Plasticizer concentrations of 30 wt% and 40 wt% were considered as the most common.

### 1.2. Starch Plasticized by Bio Ionic Liquids

**Figure 10:**
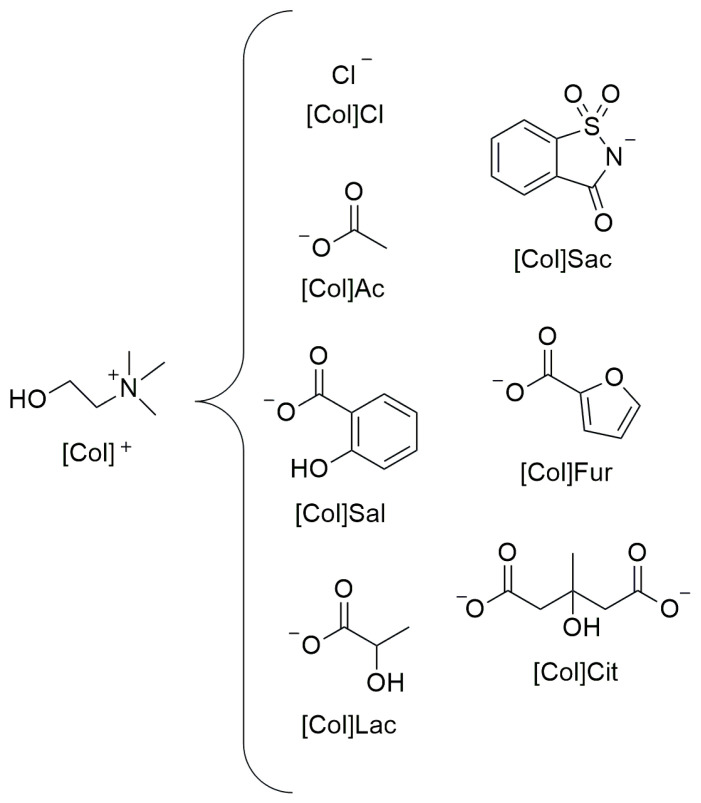
Structures of bio-ionic ILs based on choline cation considered in this review.

In 2016, Colomines et al. synthesized a series of bioionic liquids (bioILs) based on choline cation, with the aim of screening their properties as potential biobased starch plasticizers [57]. By using the solvent casting method as well, they prepared corn starch films plasticized with 30 wt% (dry basis) of choline ionic liquids by varying the anions. Starting with choline chloride ([Chol]Cl), they then employed choline acetate ([Chol]Ac), choline salicylate ([Chol]Sal), choline saccharinate ([Chol]Sac), tricholinium citrate ([Chol]Cit), cholinium furoate ([Chol]Fur), and cholinium lactate ([Chol]Lac) (Figure 10).

First, from a qualitative point of view, Colomines and the coworkers asserted that not all the bioionic liquids used were able to plasticize starch and thus have good film-forming abilities. In fact, only three ionic liquids ([Chol]Ac, [Chol]Cit, and [Chol]Lac) produced films comparable to those prepared using glycerol as a plasticizer. In particular, using [Chol]Sac failed to produce the film, while employing [Chol]Sal and [Chol]Fur resulted in brittle and less homogeneous films. The first characterization of the films concerned their crystal structure and its evolution over time performed through an XRD analysis. After successful solubilization and processing, the authors observed the disappearance of the native A-type crystalline structure characteristic of corn starch. It is important to note, however, that for no sample did they observe the emergence of the V_H_-type structure typically associated with films upon preparation. When [Chol]Ac was employed, the film exhibited the characteristic peaks of the B-type crystalline structure after just 2 weeks of storage, a behavior comparable to that of glycerol-plasticized films. In contrast, all other bioILs destroyed the starch crystalline order and promoted a highly amorphous structure, which was preserved over time for up to 8 weeks of storage. Despite the highly amorphous structure of some films, the differential scanning calorimetry analysis (DSC) revealed that the efficiency of these bioILs to lower the *T_g_* of starch appeared to be similar to conventional plasticizers, such as glycerol. Comparing these results with those of their previous work [28] on [BMIm]Cl, the authors, however, showed that the *T_g_* and water uptake values were more influenced by the type of cation, whereas the anion slightly modulated this property.

Moreover, investigating the evolution of thermo(hydro)mechanical behavior, Colomines and co-workers found that, again, the cation had a greater effect. Indeed, despite the very different *T_g_* and water content values, the mechanical behavior of the films plasticized by the choline bioILs was very similar to each other and in contrast to that containing [BMIm]Cl.

Subsequently, Decaen et al. continued the study on choline ionic liquids, selecting choline chloride ([Chol]Cl), choline acetate ([Chol]Ac), and choline lactate ([Chol]Lac) as starch plasticizers and glycerol as a reference [58].

The authors’ aim was to investigate the influence of such ionic plasticizers in starch processing methods, in particular through simple compression molding and through extrusion. In this study, the authors again investigated the effect of these ionic plasticizers on the crystal structure of starch and then performed High-Performance Size-Exclusion Chromatography coupled with Multi-Angle Laser Light Scattering and Differential Refractive Index Detection (HPSEC-MALLS-DRI), DSC, and DMTA analyses.

Again, the presence of the ionic liquids resulted in the loss of the A-type crystal structure, highlighting this efficiency for both processing techniques. However, it is interesting to note that when using compression molding or extrusion as opposed to film casting, the choline ionic liquids induced the formation of a minimal percentage of V_H_-type structure, except for [Chol]Ac. Furthermore, the authors observed that during storage for one year at 25 °C/50% RH, processing via the extrusion of these samples resulted in a progressive re-crystallization of starch with the formation of B-type crystallinity. In particular, the values obtained in the presence of [Chol]Lac and [Chol]Cl were similar, while in the presence of [Chol]Ac, the B-type re-crystallization was significantly stronger, confirming the behavior of this ionic liquid highlighted in their previous work.

The study continued with the evaluation of the macromolecular characteristics of starch samples processed through compression molding and extrusion using HPSEC-MALLS-DRI.

**Figure 11:**
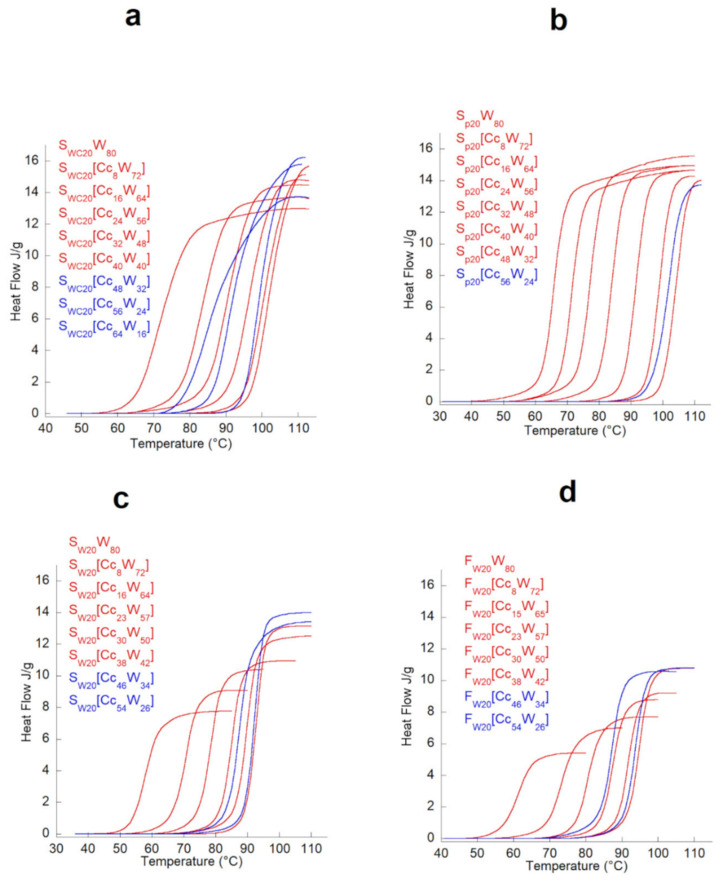
A thermal analysis of gelatinization for all the plasticizer contents studied. (**a**) Waxy-corn starch, (**b**) potato starch, (**c**) wheat starch, (**d**) wheat flour [63] © 2021 by the authors. Licensee MDPI, Basel, Switzerland. This article is an open access article distributed under the terms and conditions of the Creative Commons Attribution (CC BY) license.

The chromatograms and molecular weight distributions of corn starches obtained after compression molding showed similar patterns to those of native starch, however, with higher mean molecular weight (Mw) values for starches with all ionic plasticizers. Considering the extrusion process, the chromatogram of extruded corn starches mainly showed a peak and lower molecular weight due to the degradation of amylopectin, as already observed for extruded starches (Figure 11) [59,60] and also for starches treated with ionic liquids.

Decaen and co-workers pointed out that considering the effect of plasticizers on extruded starches, choline salts induced a greater degradation of amylopectin than glycerol during the processing process, thus explaining the higher efficiency of ionic plasticizers for starch destruction/thermoplasticization. More specifically, the authors observed that the degradation of chains was lower using [Chol]Cl than [Chol]Ac and [Chol]Lac, in contrast to what had been evidenced by Sciarini et al. [61] and thus highlighting that the use of [Chol]Cl allowed for a better compromise between efficient degradation at low specific mechanical energies and limited starch chains’ scission during extrusion. The results of the thermal characterization by DSC were in line with their previous work, obtaining similar *T_g_* values for all extruded starch samples plasticized with choline ionic liquids. The results of the thermal characterizations by DSC were in line with previous work, obtaining similar *T_g_* values for all extruded starch samples plasticized with choline ionic liquids. Finally, the results of the thermomechanical characterization showed that the nature of the cation had a greater influence on the behavior of the plasticized starch.

Decaen and collaborators have furthered and deepened the investigation into the influence of ionic plasticizers on starch processing methods, comparing the use of a micro-compounder for extrusion with Rheoplast processing [62]. In particular, they made a comparison between the use of choline-based ionic liquids ([Chol]Cl and [Chol]Ac) and those based on imidazoles ([EMIm]Cl and [EMIm]Ac). The work focuses more on a study of the processing technique, confirming the results of the previous research. However, the authors observed that imidazole-based ionic liquids were able to reduce the starch viscosity. Specifically, it was demonstrated that among various plasticizers, [EMIm]Ac emerged as the most promising ionic liquid for starch processing, as it achieved the lowest shear viscosity values while minimizing macromolecular degradation.

Finally, in 2021, Crucean et al., based on the research previously conducted by Decaen and coworkers, studied more thoroughly the influence of choline chloride on the gelatinization process of starch [63]. Evaluating the effect of [Chol]Cl on three different types of starch, the authors investigated the mechanism of gelatinization and ionic liquid penetration into starch granules through DSC and XRD techniques. The results of the thermal analysis showed that at low concentrations of [Chol]Cl, the starch underwent typical gelatinization, represented by an endothermic transition (Figure 11). Later, when the concentration of [Chol]Cl increased, the gelatinization peak shifted to higher temperatures but then decreased for all three starches. Indeed, the authors assert that the shift in gelatinization temperature toward higher values as well as the increase in total gelatinization enthalpy in the presence of [Chol]Cl could be due to the reduction in water activity in the starch/plasticizer solution, which causes an increase in the energy required for chemical and physical reactions involving water. In addition, the study of cumulative enthalpy curves showed how the type of starch, and thus of granules and their polymorphic structures, affects ionic liquid penetration. In fact, the increase in the total enthalpy of gelatinization of wheat starch at high concentrations of [Chol]Cl suggested a better organization of the ordered regions of this type of starch in the presence of the ionic plasticizer.

Finally, to understand the phenomena underlying the observed exothermic transitions, the authors performed XRD measurements in a heating cell on systems with [Chol]Cl and without. The results confirmed that the addition of the ionic plasticizer resulted in a loss of crystalline order for all types of starch at room temperature. However, when [Chol]Cl was added to the systems, the collapse of the remaining starch crystal structures was observed at much higher temperatures than in systems without a plasticizer. This result confirmed that the addition of the ionic liquid caused a reorganization of the internal structure of the starch grains.

## 2. Conclusions

Ionic liquids are ideal for starch processing, in particular offering an interesting alternative to conventional plasticizers of this polysaccharide. Over the past few years, research is increasingly investigating the interaction mechanisms between starch and ILs, expanding the potential of this biopolymer in numerous fields of application. Indeed, thanks to the properties of ILs, starch-based materials are gaining more and more interesting characteristics, competing with conventionally plasticized films from glycerol. Therefore, it has become important to understand how ionic plasticizers interact with the starch matrix. All the scientific contributions investigated in this review showed that FT-IR spectroscopy, X-ray diffraction analysis (XRD), differential scanning calorimetry (DSC), and mechanical tests are techniques capable of obtaining information on starch/IL interactions. First, studies evaluated the effect of imidazolium ILs and their plasticizer ability, starting with 1-allyl-3-methylimidazolium chloride ([AMIm]Cl) and varying the cation with 1-butyl-3-methylimidazolium ([BMIm]Cl). The results showed that these ILs were able to decrease the ability of starch to form hydrogen bonds compared to glycerol, and furthermore, different cations correspond to different interaction mechanisms with starch. Indeed, the ability of [BMIm]Cl to interact with the oxygen and hydrogen of the same C-O-H groups was revealed, thus preventing starch chains from forming interchain hydrogen bonds. Finally, the good plasticizing abilities of [BMIm]Cl led to the exploration of the possibility of blending thermoplastic starch with other polymers and to the exploration of new sustainable processing technologies and green approaches.

Subsequently, expanding the research to different anions, [EMIm]Ac was found to be one of the ideal ILs for starch plasticization. Indeed, it interacts well with all starch structures, regardless of amylose and amylopectin content and processing technique.

Finally, the investigation also highlighted the importance of the water contribution in starch plasticization processes through ILs, prompting research on finding a proper formulation that balances the contribution of ILs with that of water.

On the other hand, the search for more environmentally friendly alternatives has led to the exploration of biobased plasticizers, with particular interest in choline-based ionic liquids. Not all ionic liquids derived from choline have been found to have good plasticizer properties. In fact, in three cases, namely [Chol]Cl, [Chol]Ac, and [Chol]Lac, TPS films were prepared following an industrial perspective by selecting extrusion and thermocompression as processing techniques [64,65]. Assured of the familiarity of glycerol with these techniques [66], a truthful comparative study of the physico-chemical properties of the latter with those based on the reference plasticizer glycerol was possible. Moreover, the comparative survey between bioILs and imidazolium ILs was conducted. It is interesting to note that research has shown a greater influence of the cation on starch/bioIL interactions than imidazolium IL ones, especially if *T_g_* and water uptake were taken as reference parameters. In both cases, however, the good plasticizer properties of the acetate anion have been observed.

Ongoing research and development efforts are moving towards the exploration of new ILs, with the new family of dicationic ionic liquids also being explored. Furthermore, as environmental concerns drive society to replace current plasticizers with more environmentally friendly alternatives, the adoption of natural-based plasticizers is expected to grow.

## Figures and Tables

**Table 1:** Properties of plasticized starch, referred to as 30% or 40%, as IL weight percentage with respect to starch (indicated in parentheses).

IL	Starch Crystallinity	*T_g_* (°C) ^a^	*T_deg_* (°C) ^b^	Young Modulus (MPa)	Tensile Strength (MPa)	Elongation at Break (%)	σ (S cm^−1^) ^c^	Conditions ^d^	Ref.
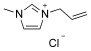	A-type	-	-	-	-	-	10^−1.6^ (30%)	RH = 50% SC	[22]
		−49 (30%) −62 (40%)	-	-	-	-	-	RH = 35% TC	[33]
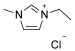	A-type	−28 (30%) −59 (40%)	-	-	-	-	-	RH = 35% TC	[33]
		-	261 (40%)	14 (40%)	1 (40%)	22 (40%)	10^−5^ (40%)	RH = 50% SC	[52]
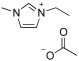	A-type	−28 (30%) −59 (40%)	-	-	-	-	-	RH = 35% TC	[33]
	B-type	-	266 (30%)	50 (30%)	5 (30%)	20 (30%)	-	RH = 52% TC	[34]
	A-type	-	266 (30%)	50 (30%)	5 (30%)	65 (30%)	-	RH = 52% TC	[34]
	A-type	-	265 (30%)	150 (30%)	10 (30%)	80 (30%)	-	RH = 33% TC	[38]
	B-type	-	260 (30%) 260 (40%)	105 (30%) 50 (40%)	6 (30%) 4 (40%)	49 (30%) 43 (40%)	-	RH = 50% SC	[51]
	A-type	-	269 (40%)	53 (40%)	3 (40%)	42 (40%)	10^−6^ (40%)	RH = 50% SC	[52]
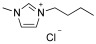	A-type	−13 (30%)	-	0.5 (30%)	0.6 (30%)	392 (30%)	10^−4.6^ (30%)	RH = 50% TC	[25]
		−60 (30%) −87 (40%)	-	-	-	-	-	RH = 35% TC	[33]
		-	-	-	1.5 (30%)	316 (30%)	-	RH = 33% TC	[45]
		-	255 (30%)	-	0.8 (30%)	350 (30%)	-	RH = 52% TC	[45]
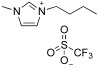	A-type	−22 (30%)	-	-	-	-	10^−4^ (30%)	RH = nr ^e^ SC	[32]

^a^ Glass transition temperature. ^b^ Degradation temperature. ^c^ Conductivity. ^d^ RH: Relative humidity. TC: Thermocompression. SC: Solution casting. ^e^ nr: not reported.

## Data Availability

No new data were created or analyzed in this study. Data sharing is not applicable.
